# Apoptosis in eosinophilic nasal polyps treated *in vitro* with Mitomycin C

**DOI:** 10.1590/S1808-86942012000300007

**Published:** 2015-10-14

**Authors:** Cláudia Pena Galvão dos Anjos, Anilton Cesar Vasconcelos, Paulo Fernando Tormin Borges Crosara, Gustavo Coelho dos Anjos, Celso Gonçalves Becker, Roberto Eustáquio Santos Guimarães

**Affiliations:** a MD, MSc (Otorhinolaryngologist).; b MD, PhD (Professor in the Department of Pathology of the Biologic Sciences Institute of the Federal University of Minas Gerais).; c MD, PhD (Associate Professor in the Department of Ophthalmology, Otorhinolaryngology, and Speech and Hearing Therapy of the Medical School of the Federal University of Minas Gerais).; d MD, MSc (Otorhinolaryngologist).; e MD, PhD (Associate Professor in the Department of Ophthalmology, Otorhinolaryngology, and Speech and Hearing Therapy of the Medical School of the Federal University of Minas Gerais).; f Professor (Associate Professor in the Department of Ophthalmology, Otorhinolaryngology, and Speech and Hearing Therapy of the Medical School of the Federal University of Minas Gerais). Faculdade de Medicina da Universidade Federal de Minas Gerais.

**Keywords:** apoptosis, mitomycin, nasal polyps

## Abstract

The etiopathogenesis of eosinophilic nasal polyps is yet to be explained. Eosinophils are key components in the inflammatory infiltrate and are related to the perpetuation of the inflammatory process in chronic rhinosinusitis with nasal polyps.

**Objective:**

This paper aims to evaluate the *in vitro* action of mitomycin upon the apoptotic index of nasal polyps.

**Materials and Methods:**

This is a self-paired prospective experimental study using biopsy fragments from 15 patients with eosinophilic nasal polyps. Biopsy fragments were divided into two groups. In the case group, the fragments were treated with 400 µg/ml of mitomycin for five minutes. The control group fragments were treated with culture medium. The pair of fragments contained in the two first compartments - control and case - were immediately sent to the histopathologist. The other pair of samples containing control and case fragments was incubated for 12 hours. The fragments were then taken to the histopathologist for testing. The apoptotic index was determined by the morphometry in hematoxylin and eosin staining and DNA fragmentation analysis (TUNEL reaction).

**Results:**

The comparison between the two groups showed a statistically significant difference (*p* < 0,001) in the apoptotic index of the 12-hour incubated cultures.

**Conclusion:**

Mitomycin acts *in vitro* upon the eosinophilic nasal polyps inducing the rise of the eosinophilic apoptotic index.

## INTRODUCTION

Eosinophilic Nasosinusal Polyposis is a chronic inflammatory proliferative affection of the nasal mucosa and paranasal sinuses. Eosinophilic Nasosinusal Polyposis triggers different degrees of obstruction of the upper respiratory tract and both atopic patients and the general population suffer from it^1^, ^2^. Nasal polyposis is considered part of a subdivision of chronic rhinosinusitis since it appears impossible to clearly differentiate both conditions^3^.

It is usually bilateral and has higher incidence in the fourties affecting 2.7% of the population, the majority of cases being men (with a proportion rate of 2.2/1). It is associated with asthma, eosinophilic non-allergic rhinitis, aspirin intolerance, eosinophilic fungal sinusitis, and Churg-Strauss syndrome. The incidence rate of Eosinophilic Nasosinusal Polyposis is similar in both atopic patients and the general population, and its occurrence rises with the concomitant of bronchial asthma^4^, ^5^.

Although the mechanisms of Eosinophilic Nasosinusal Polyposis development are yet to be fully understood, it is known that the inflammatory response is involved in its formation process. It is assumed that the nasal polyps could be the final stage of a complex inflammatory process which is the result of multiple etiologies. The eosinophils and the structural polyp cells produce cytokines that keep both the ongoing inflammatory process and the recruitment of new eosinophils. Cytokines such as Interleukin-5 (IL-5) and granulocyte-macrophage colony-stimulating factor (GM-CSF) prolong the life span of eosinophils and improve their presence in the polypoid tissue, thus lowering the apoptosis index of these cells^6^. The detection of the vascular endothelial growth factor in cases of chronic sinusitis with polyps was linked to the induction of cell proliferation and inhibition of apoptosis of the epithelial cells evaluates in such cases^7^.

The corticosteroids are the main therapeutic clinic option for the nasosinusal polyposis due to its role in diminishing the inflammatory processes. It was possible to show the action of the corticosteroids over the apoptotic index of the cells of the stroma inflammatory infiltrate from Eosinophilic Nasosinusal Polyposis^8^, ^9^, ^10^. The growth of the BAX gene expression (pro-apoptotic) in eosinophilic nasal polyps raised the apoptosis index induced by the treatment with glicocorticoids^11^.

The quest for other therapeutic options has led to the study of the effect of drugs that can act in the inflammatory process cells. The aim would be to interrupt the pathophysiologic process that promotes and perpetuates the relapse in the nasosinusal polyposis occurrence. Mitomycin-C is an antineoplastic and antibiotic drug extracted from *Streptomyces caespitosus* that has been used as an antiproliferative agent^12^. In otorhinolaryngology the application in humans have focused mainly on the action of the drug in the reduction of sinechiae in endoscopic nasal surgery^13^ as well as in laryngeal surgery^14^. Both human and animal applications confirm the safety of topical use of mitomycin-C^15^. Works that investigate the action of mitomycin-C in eosinophilic nasal polyps are scarce. It was possible to show the action of *in vivo* and *in vitro* mitomycin-C in diminishing the levels of both interleukin-5 and also granulocyte-macrophage colony-stimulating factor (GM-CSF) in eosinophilic nasal polyps. These oligoproteins diminish the rate of apoptosis in eosinophilic nasal polyps and prolong the survival of eosinophils^16^, ^17^, ^18^.

The aim of this study is to evaluate the actions of *in vitro* mitomycin-C in the eosinophilic nasal polyp's eosinophils through autopaired experimental study with evaluation of the apoptotic index.

## MATERIALS AND METHODS

This study has been submitted to and approved by the Research Ethics Committee under ETIC 484/08. Fifteen samples of Eosinophilic Nasosinusal Polyposis patients with eosinophils infiltration equal or higher than 40%^19^, ^20^, ^21^ were studied. The research did not consider patients with non-eosinophilic polyposis or Eosinophilic Nasosinusal Polyposis patients with ongoing infection processes or under corticosteroids or histamine antagonist over 30 days before the study was actually carried out. Patients with septal deformities or anatomic variations have also been excluded. The major symptoms displayed by the patients were: anosmia, hyposmia, nasal obstruction, and post-nasal drip. All patients were advised to seek surgery. At first the polyps were extracted through biopsy of the medium meatus and put in 10% formol solution. The samples were then proceeded to histopathologic exams with hematoxylin and eosin staining so as to characterize eosinophilia. Following that, the included patients underwent another biopsy. The samples of this biopsy were put in a three-orifice plate, each containing 250µl of culture medium RPMI 1.640^22^. The fragments contained in the first orifice were immediately prepared for histopathology and hence denominated zero hour control (0h). The other orifices constituted the control group (12 hours) and experimental group (12 hours). A 400 µg/ml dose of mitomycin-C was administered to the experimental group for five minutes^23^. After that, the cultures were washed with RPMI culture medium. The control group underwent the same procedures, but with the use of only RPMI 1640 culture medium. Fragments of both groups (control and experiment) were incubated at 37° C (98° F) and 0.5% CO_2_ for 12 hours. After that period, they were submitted to histopathology exams^24^. The samples of 15 patients were subjected to routine histopathology preparation (hematoxylin and eosin stain) for evaluation of the apoptotic index through morphometry. A random sub-sample (n = 6 patients) underwent the TUNEL reaction for evaluation of DNA fragmentation.

### Analysis of the apoptotic index through histopathology

For the analysis of the apoptosis index through hematoxylin and eosin stain, each blade had their eosinophils count in immersion oil at 100X magnification and their apoptosis index calculated. The figure was calculated by dividing the sum of eosinophils in apoptosis by the global sum of counted eosinophils^25^.

**Figure 1:**
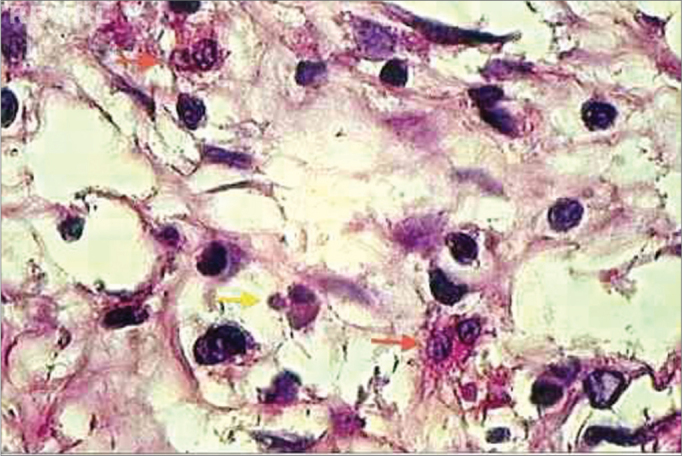
histopathology of eosinophilic nasosinusal polyp (control group) incubated for 12 hours. (H. and E. 1000x).

**Figure 2:**
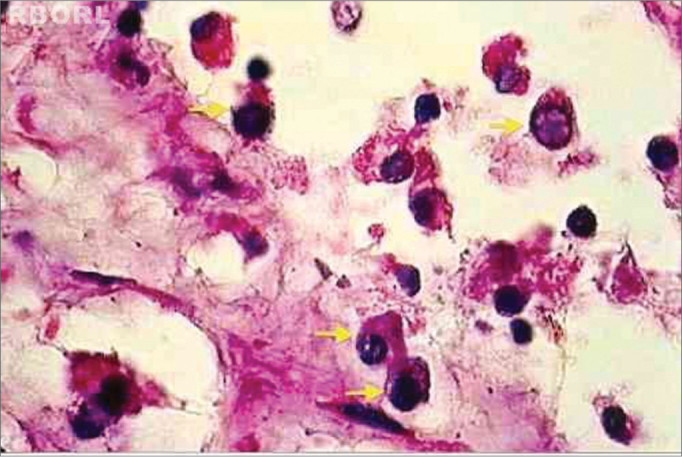
Histopathology of eosinophilic nasosinusal polyp treated with mitomycin C and incubated for 12 hours (H. and E. 1000x). It is possible to identify the compatible alterations with eosinophils apoptosis in most of the cells (yellow arrows).

For characterizing an eosinophil in apoptosis, several aspects were taken into account, namely: anoikis (cell retraction with loss of adherence between the adjacent cells); cytoplasmic condensation; nuclear condensation (chromatin condensation, with possible compaction of chromatin against karyotheca, displaying “crescent” figures); nuclear and cytoplasmic fragmentation and formation of apoptotic bodies (Figures 1 and 2)^25^, ^26^, ^27^. Microscopic review was performed by two different searchers.

### Analysis of the apoptotic index through DNA fragmentation (TUNEL reaction)

**Figure 3:**
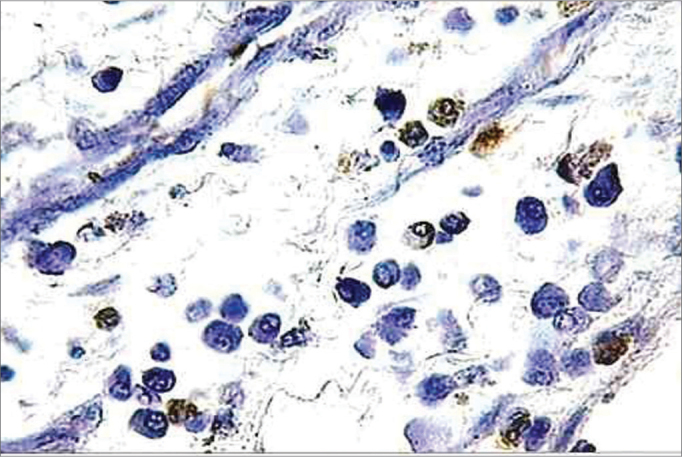
Histopathology of eosinophilic nasosinusal polyp (control group) incubated for 12 hours. (TUNEL 400x)

**Figure 4:**
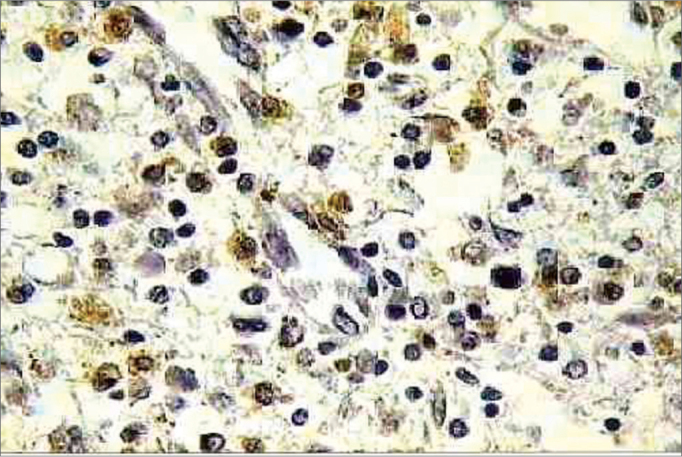
Histopathology of eosinophilic nasosinusal polyp treated with mitomycin C and incubated for 12 hours (TUNEL 400x).

The occurrence of apoptosis was evaluated through the observation of genomic DNA fragmentation through TUNEL reaction. The procedure was carried out with the use of a commercial *in situ* genoma fragmentation detection kit (*Klenow FragEl DNA fragmentation*. *Detection Kit* - In QIA21 Catalog; *Calbiochem /Oncogene*- *internet address:* http://calbiochem.com/). The proteinase K span was set to 20 minutes. The endogenous peroxidase was deactivated with 3% hydrogen peroxide for 5 minutes. The blades were washed with PBS (*Phosphate Buffer Saline*) and immersed in an equilibrium buffer solution. The samples were covered with the TdT enzyme (terminal deoxynucleotidyl transferase) and deoxynucleotides (labeled and non-labeled) and incubated in humid atmosphere at 37°C (98°F) for 2 hours. Then, they were washed in TBS 1X and covered in stop buffer (EDTA 0.5 M, ph = 8) for 5 minutes. Once again were the blades washed in TBS 1X and covered in blocking buffer for 10 minutes. They were kept in peroxidase-conjugated streptavidin diluted in blocking buffer and incubated in a humidified chamber at 37°C (98°F) for 30 minutes. The blades were washed in TBS 1X again and kept in DAB (Diaminobenzidine) for 6 minutes, washed in distilled water, counter stained with hematoxylin, and mounted. Positive and negative controls were used in all reactions (Figures 3 and 4). Eosinophil count was recorded by one searcher.

The eosinophils count was done at 40X magnification with the apoptosis index calculated. The apoptosis index was calculated by dividing the sum of eosinophils in apoptosis by the global sum of counted eosinophils.

### Statistical analysis

The computer programs used for statistical analysis were *Statistical Package for Social Science* (SPSS), version 17.0 (SPSS. Inc, Chicago, IL, USA) and *The R Project for Statistical Computing* (GNU General Public License); version 2.9.1.

The results achieved were submitted to the Kolmogorov-Smirnoff and Shapiro-Wilk tests for verification of normality. The analyzed data were submitted to the Kruskall-Wallis test for comparison of apoptotic indexes, followed by the Wilcoxon post-test with Bonferroni correction. A significance level of 5% was considered.

## RESULTS

**Figure 5:**
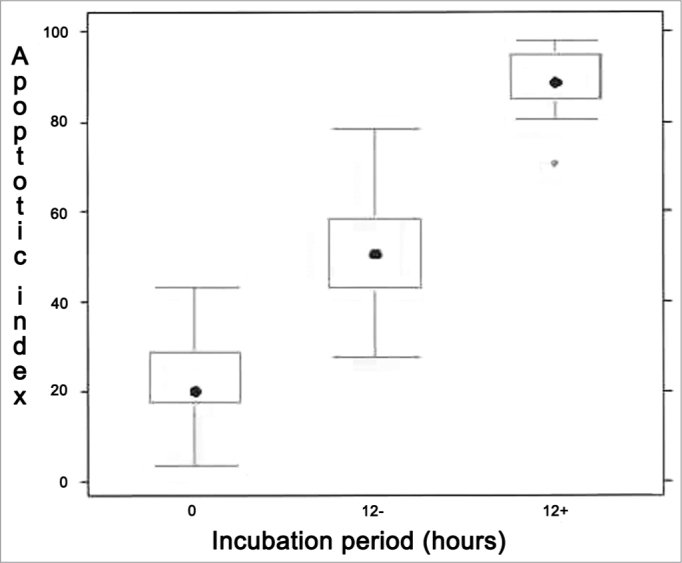
Analysis of apoptotic index through morphology (hematoxylin and eosin stain. 1000x magnification). Kruskall Wallis test: *p* < 0,001 for all patients. Wilcoxon post-test with Bonferroni correction: *p* < 0,001 for all patients/comparisons, except for both zero marks in which *p*-value= 0.1724. “0” refers to zero hour incubation period; “12-” is 12 hour incubation with no treatment whatsoever and “12+” is 12 hour incubation with Mitomycin-C.

1.214 frames were captured and analyzed in the apoptosis index evaluation through morphometry. The comparison between the 0h control, 12h control, and 12h experimental groups has shown significant difference between the three experiments (*p* < 0,001), as shown in Figure 5.

**Figure 6:**
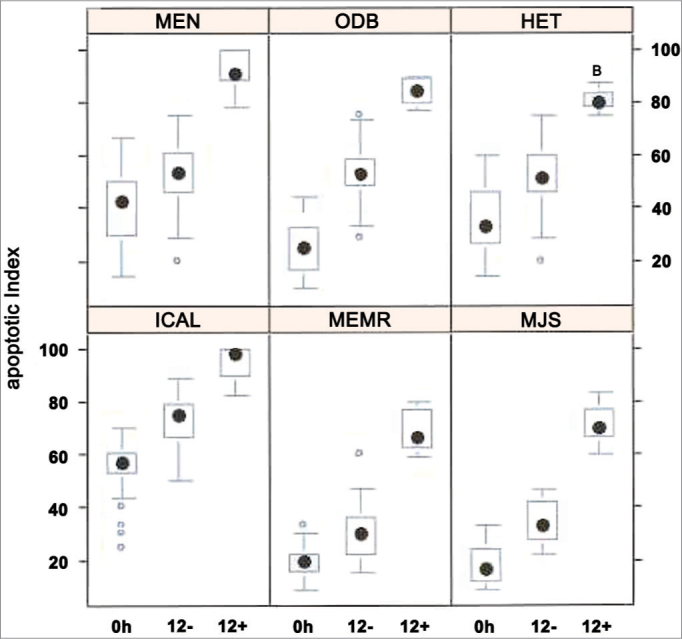
Apoptotic index (n = 6 patients) evaluated through TUNEL reaction. Kruskall Wallis test: *p* < 0,001 for all patients. Mann-Whitney post-test with Bonferroni correction: *p* < 0,001 for all patients/comparisons.

In the apoptotic index analysis through TUNEL reaction, 645 frames were captured and analyzed at 400x magnification. All patients displayed significant difference in the three experiments: 0h control group, 12h control group, and 12h experimental group (*p* < 0,001).The positive TUNEL reaction was highlighted for all groups. The nuclei of cells in apoptosis were clearly distinguished by their brownish color. It was also possible to notice positive TUNEL reaction in other inflammatory infiltrate cells of the eosinophilic nasal polyps. The individual result of the apoptotic index of the sub-samples of 6 patients *in vitro* can be evaluated in Figure 6.

## DISCUSSION

Eosinophils are the main kind of cells related to the Eosinophilic Nasosinusal Polyposis physiopathology. It is known that they prolong the life span in the inflammatory infiltrate of Eosinophilic Nasosinusal Polyposis patients^28^. The removal of eosinophils from the polypoid tissue occurs through apoptosis^29^. The number of those cells in apoptosis in the inflammatory infiltrate in Eosinophilic Nasosinusal Polyposis patients is lower than that found in healthy tissues^16^. Eosinophils are able to produce a great amount of substance through which they can damage tissues and perpetuate the inflammatory process^30^. Although the Eosinophilic Nasosinusal Polyposis physiopathology is yet to be fully understood, it is clear that the inflammatory response is involved in its formation process. Cytokines such as IL5 and GM-CSF prolong the eosinophils life span and therefore their presence in the polypoid tissue, thus lowering the apoptosis level of these cells. These cytokines can be produced from an autocrine process. So the inhibition of the apoptosis process would slow the removal of eosinophils of the tissue, promoting in an indefinite manner the Eosinophilic Nasosinusal Polyposis inflammatory process^6^, ^26^, ^31^, ^32^. Therefore, in this current study, there was a conscious choice for the evaluation of the apoptotic level of the eosinophils of the inflammatory infiltrate of Eosinophilic Nasosinusal Polyposis patients.

Mitomycin C has been used in preventing sinechiae and stenosis in endoscopic nasal surgery. The topic use of mitomycin-C is safe^13^. Studies on the substance as well as eosinophilic nasal polyps are scarce. The reduction of IL-5 and GM-CSF levels in eosinophilic nasal polyps *in vitro* suggests indirect mechanism of mitomycin-C in the eosinophilic nasal polyp's eosinophils apoptosis^17^, ^18^. In diminishing the expression of antiapoptotic proteins, Mitomycin-C would promote an imbalance between the proteins which regulate the apoptosis, thus resulting in the liberation of the apoptotic cascade.

In this study, mitomycin-C was the only variable in the manipulation of the control and experimental groups. On comparing the 12h control group and the 12h experimental group, it was noticeable that the growth of the apoptotic index in such groups can be partly explained by the cultures' incubation period. The comparison between control and experimental in different incubation periods makes it possible to attribute the difference of the apoptotic index to the effect of mitomycin-C. Such results confirm the finding of specialized literature in what regards the actions of mitomycin-C in the inductionof the eosinophilic nasal polyp's eosinophils apoptosis *in vitro*.

One of the aims of this study was to estimate the biologic event known as apoptosis. Two different methods were used: routine histopathology (hematoxylin and eosin stain) and immunohistochemical reaction (TUNEL reaction). It was possible to identify the compatible alterations with the apoptosis evolution through morphometry: cytoplasmic condensation, nuclear condensation, loss of cellular contact, and formation of apoptotic bodies. The TUNEL reaction is a standard method for the evaluation of apoptosis. This method allows for highlighting the genomic DNA fragmentation that prematurely occurs in the apoptosis process. It's a decisive stage of the process since from that point on it becomes irreversible. The formation of apoptotic bodies inhibits both the releasing of internal cellular constituents and the activation of the inflammatory mediators. Apoptosis results in the resolution of the inflammatory process, quite differently from the necrotic process which allows for premature permeabilization of the cell membrane^33^. Since the estimative of a biologic event was one of the objectives of this study, the use of different methods makes it more reliable. This study lags comparison between Mitomycin -C treatment and Glucocorticoids treatment. Glucocorticoids have potent anti-inflamatory action and are the most common treatment for nasal polyps. It was shown the effect of glucocorticoidsover the apoptotic index of the cells of the stroma inflammatory infiltrate from Eosinophilic Nasosinusal Polyposis.

The expression of different proteins with antiapoptotic action on the onset of Eosinophilic Nasosinusal Polyposis was shown in several studies; these only come to reaffirm the importance of the alteration in the apoptotic mechanisms in the nasal polyposis physiopathology^34^, ^35^, ^36^, ^37^. One of these mechanisms of actions of mitomycin-C is likely to be the lowering of the interleukins (IL-5 and GM-CSF) with reduction of the expression of proapoptotic proteins^17^, ^32^. So Mitomycin-C enhances the suspension of antiapoptotic mechanism, thus making it possible for the apoptotic index in eosinophilic nasal polyp's eosinophils to grow. The eosinophils apoptosis can make for the resolution of the Eosinophilic Nasosinusal Polyposis inflammatory process. The analysis of samples though TUNEL reaction has also spotted fibroblasts, macrophages, and specially lymphocytes in the apoptotic process. The brownish color of these cells was more evident in the 12h experimental group. It is assumed that mitomycin-C also acts upon the other inflammatory infiltrate cells as well as upon the nasal polyp's stroma. Further studies will be able to look into the action of mitomycin-C in the other eosinophilic nasal polyp's cells.

Mitomycin-C represents a prospective option in the Eosinophilic Nasosinusal Polyposis treatment for the future and the study of its clinic administration allows for a more in-depth knowledge of the apoptotic tracts involved in the Eosinophilic Nasosinusal Polyposis physiopathology.

## CONCLUSION

Therefore it is concluded that mitomycin-C acts *in vitro* upon eosinophilic nasal polyps hence inducting the growth of the apoptotic index of *in vitro* eosinophils. This study is *in vitro* and may not replicate what happens *in vivo*. Further studies will be necessary to study the action of mitomycin-C in eosinophilic nasal polyp *in vivo*.
